# Surgical outcomes of robotic thyroidectomy vs. conventional open thyroidectomy for papillary thyroid carcinoma

**DOI:** 10.1186/s12957-016-0929-y

**Published:** 2016-07-09

**Authors:** Jeong Nam Cho, Won Seo Park, Sun Young Min, Sang-Ah Han, Jeong-Yoon Song

**Affiliations:** Department of Medicine, Graduate School, Kyung Hee University, Seoul, Republic of Korea; Department of Surgery, Kyung Hee University Medical Center, Kyung Hee University School of Medicine, Seoul, South Korea; Department of Surgery, Kyung Hee University Hospital at Gangdong, Kyung Hee University School of Medicine, Seoul, South Korea; Kyung Hee University Hospital, 23 Kyungheedae-ro, Dongdaemun-gu, Seoul, 02447 Republic of Korea; Department of Thyroid-Endocrine Surgery, Kyung Hee University Hospital, 23 Kyungheedae-ro, Dongdaemun-gu, Seoul, 02447 Republic of Korea; Kyung Hee University Hospital at Gangdong, 892 Dongnam-ro, Gangdong-gu, Seoul, South Korea

**Keywords:** Robotic surgical procedure, Thyroid neoplasm, Thyroidectomy, Treatment outcome

## Abstract

**Background:**

The purpose of this study was to compare the surgical outcomes of robotic thyroidectomy (RT) using bilateral axillo-breast approach (BABA) with conventional open thyroidectomy (OT) in papillary thyroid carcinoma patients.

**Methods:**

Between January 2009 and December 2013, 815 patients who had received thyroidectomy for papillary thyroid carcinoma were enrolled. Of these, 126 patients received RT and 689 patients underwent OT. Age, gender, body mass index, extent of surgery, tumor size, multiplicity, bilaterality, extrathyroidal extension, and tumor stage were used for the propensity score matching analysis. One hundred and nine patients were selected in each group, and surgical outcomes were compared between the two groups.

**Results:**

The RT group showed a significantly longer operating time (290.6 ± 74.4 vs. 107.9 ± 30.8 min, *P* < 0.001). However, the mean hospital stay after surgery (3.6 ± 0.8 vs. 3.4 ± 1.2 days, *P* = 0.293), postoperative complication rates (major and minor, *P* = 0.754 and *P* = 0.852), and pain score (postoperative day, *P* = 0.669; postoperative day 1, *P* = 0.952) were comparable between the two groups. There was no difference in the number of metastatic lymph nodes, but the mean number of retrieved lymph nodes in the RT group was lesser than that in the OT group (3.5 ± 3.5 vs. 5.3 ± 5.2, *P* = 0.002).

**Conclusions:**

Robotic thyroidectomy via the BABA may be a safe and acceptable surgical technique. But, further development that resolves the limitation of central node dissection is needed.

## Background

Since the time minimally invasive endoscopic thyroid surgery has been introduced [1], various methods have been developed to improve accessibility and cosmetic satisfaction. Thereafter, minimally invasive surgery was extended to the field of papillary thyroid carcinoma (PTC) and many studies have reported favorable surgical outcomes including outstanding cosmetic results [2–4]. But, endoscopic thyroidectomy has several limitations such as restricted motion of surgical instruments and two-dimensional camera view. These limitations were reduced by using the da Vinci S robotic system (Intuitive Surgical, Sunnyvale, CA) that offered several advantages to the surgeon with a highly magnified three-dimensional view, fine motor scaling, tremor-free, and endo-wrist function [5, 6]. A number of studies showing promising surgical outcomes of robotic thyroidectomy (RT) have been published [7–26]. But, there has been no randomized study for RT and any other robotic surgery, and it may be difficult to perform such a study in the near future because of the fundamental limitation about the cost issues. In particular, the issue concerning the oncological safety of RT in PTC patients is debatable and there is no worldwide consensus on the definite indication for RT as yet. Therefore, we analyzed the results of RT in our hospital for the last 5 years and compared the surgical and oncological safety of RT with conventional open thyroidectomy (OT) for analyzing the feasibility of RT in PTC patients.

## Methods

**Fig. 1:**
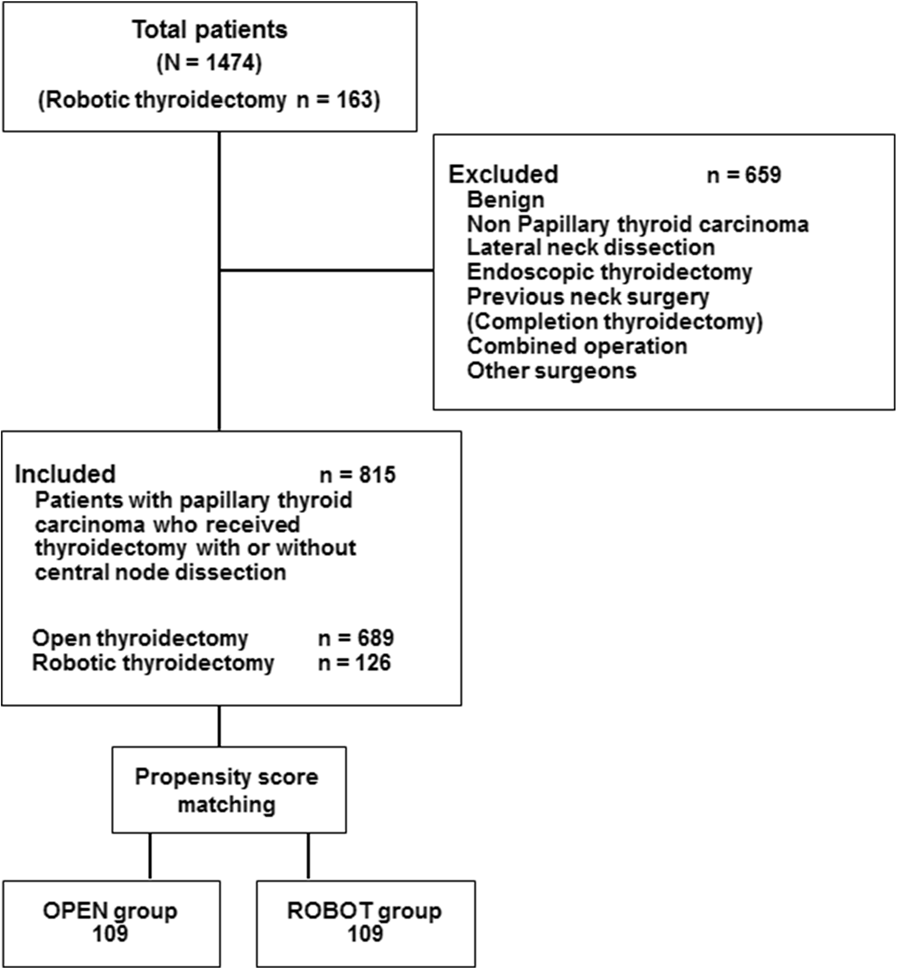
Patient selection

This retrospective cohort study was carried out between January 2009 and December 2013 in the Department of Surgery, Kyung Hee University Medical Center, Seoul, South Korea. We included 815 PTC patients who underwent thyroidectomy with or without central node dissection (CND). Of these, 689 patients received conventional OT and 126 patients underwent RT using the bilateral axillary breast approach (BABA) method (Fig. 1).

The decision regarding the operation methods, whether robotic or conventional open thyroidectomy, was made based on the patients’ preferences. Randomization of operative methods was not possible because the cost of robotic surgery is not reimbursed by national health insurance; hence, the hospital cost for RT is two to three times higher.

We conducted a propensity score matching analysis to reduce treatment selection bias and potential confounding effects [27]. We selected the following 10 factors that could affect the surgical outcomes: age, sex, body mass index (BMI), extent of surgery, extent of lymph node dissection, tumor size, multiplicity, bilaterality, extrathyroidal extension, and cancer stage [28]. One hundred and nine patients were selected in each group, and surgical outcomes were compared between the two groups.

RT was not recommended in cases with tumors >4 cm or clinically evident lateral lymph node metastasis or tumors located on the dorsal aspect of the thyroid with suspicion of invasion into adjacent organs such as the esophagus, trachea, or recurrent laryngeal nerve.

Unilateral or bilateral CND was conducted for prophylactic or therapeutic purposes. Bilateral CND was performed for bilateral tumors or in patients with contralateral suspicious lymph node enlargement on preoperative evaluation or intraoperative gross morphology. Lobectomy without CND was performed only when there was a single tumor <5 mm without extrathyroidal extension.

All patients were diagnosed with PTC or suspicious for PTC by fine needle aspiration cytology. We performed ultrasonography (US), computed tomography (CT), and thyroid function tests in all patients.

### Surgical outcomes

Details of surgery such as operation time, status of parathyroid glands, and postoperative complications were compared between the two groups. Transient hypocalcemia was defined as the patients who received calcium replacement to treat hypocalcemic symptoms and who had a serum parathyroid hormone (PTH) level <13 pg/mL regardless of their serum calcium levels. We measured the PTH level at postoperative day (POD) 2. The patients who had hoarseness with vocal cord palsy confirmed by laryngoscopy were defined as having transient hoarseness. Permanent hypocalcemia was defined as PTH level <13 pg/mL and the need for calcium or vitamin D supplements for more than 6 months after thyroidectomy. Permanent recurrent laryngeal nerve palsy was diagnosed when they lasted over 6 months.

A drain was placed in the thyroid bed, and it was removed when the amount of drainage was less than 40 mL in a day. Postoperative pain was evaluated using an 11-point visual analog scale (VAS). Pain score was checked at 1 h after surgery and once in a day until discharge. Hospital cost was determined based on the inpatient charges. The evaluation of an abnormal sequelae or complication was routinely carried out at 2 weeks after the operation and continued until 1 year after the operation in an outpatient setting. Radioactive iodine (RAI) ablation therapy was performed on the basis of cancer stage and risk factors, according to the American Thyroid Association (ATA) guidelines [29]. TSH-stimulated thyroglobulin (Tg) levels in patients who received radioiodine therapy were analyzed to compare surgical completeness between the two groups. TSH-stimulated Tg levels were checked after withdrawal of levothyroxine or injection of recombinant human thyrotropin, while TSH levels were >30 μIU/mL. The stimulated Tg and TSH levels were measured on the day of RAI ablation therapy before RAI administration. The RAI dose ranged from 30 mCi 131-I to 150 mCi 131-I. Patients received RAI ablation therapy after 8–12 weeks after thyroidectomy.

### Surgical procedures

All surgeries were performed by a single surgeon. We used the BABA technique that was introduced by Lee et al. [5] for RT. For the detailed procedure, please refer to the bibliography [5]. The only difference was that we injected normal saline instead of diluted epinephrine (1:200,000) for flap elevation.

### Statistical analysis

We used the Kolmogorov-Smirnov test to assess normality of data distribution. Baseline clinicopathologic characteristics between the OT and RT groups were compared by using the Fisher’s exact test for categorical variables and the independent sample *t* test or Mann-Whitney *U* test for continuous variables. After propensity score matching, the two groups were compared in terms of baseline clinicopathologic characteristics and surgical outcomes by using the McNemar test for categorical variables and the paired *t* test for continuous variables. All statistical tests were two-sided, and a *P* value <0.05 was considered statistically significant. Statistical analysis was performed using SPSS® version 19.0 (IBM Co., Armonk, NY, USA). The study protocol was approved by our Institutional Review Board.

## Results

### Baseline characteristics of the study groups before cohort matching

Table 1 shows the baseline clinicopathologic characteristics of the two groups before propensity score matching. The mean age was lower in the RT group than that in the OT group (39.86 ± 10.29 years vs. 52.15 ± 12.06 years, *P* < 0.001). The BMI was lower in the RT group (23.40 [range, 15.45–37.77] vs. 24.45 [range, 17.10–37.79], *P* = 0.001). The proportions of stage III disease and total thyroidectomy were significantly lower in the RT group than that in the OT group (*P* < 0.001, *P* = 0.027).

**Table 1:** Baseline characteristics of patients before propensity score matching

Variables	Open group	Robot group	*P* value
	*n* = 689	*n* = 126	
Age, years (mean ± SD)	52.15 ± 12.06	39.86 ± 10.29	< 0.001
Gender			0.351
Male (%)	110 (16.0)	16 (12.7)	
Female (%)	579 (84.0)	110 (87.3)	
BMI (median (range))	24.45 (17.10–37.79)	23.40 (15.45–37.77)	0.001
Extent of surgery			0.027
Total thyroidectomy (%)	583 (84.6)	99 (78.6)	
Subtotal thyroidectomy (%)	1 (0.1)	2 (1.6)	
Lobectomy (%)	105 (15.2)	25 (19.8)	
Lymph nodes dissection			0.400
None (%)	67 (9.7)	21 (16.7)	
Unilateral (%)	461 (66.9)	83 (65.9)	
Bilateral (%)	161 (23.4)	22 (17.5)	
No. of metastatic lymph nodes (mean ± SD (range))	0.90 ± 2.07 (0–19)	0.70 ± 1.54 (0–9)	0.611
No. of retrieved lymph nodes (mean ± SD (range))	5.12 ± 4.52 (0–35)	3.35 ± 3.48 (0–17)	< 0.001
Tumor size (cm, median (range))	0.76 (0.1–4.7)	0.74 (0.2–2.5)	0.389
Multiplicity (%)	201 (29.2)	31 (24.6)	0.334
Bilaterality (%)	157 (26.9, 157/584)	23 (22.8, 23/101)	0.294
Extrathyroidal extension (%)	281 (40.8)	45 (35.7)	0.323
Stage (%)			< 0.001
I	405 (58.8)	109 (86.5)	
II	6 (0.9)	0 (0)	
III	278 (40.3)	17 (13.5)	
IV	0 (0)	0 (0)	

### Baseline characteristics of the study groups after cohort matching

Table 2 shows the baseline clinicopathologic characteristics of the two groups after propensity score matching. After cohort matching, 109 pairs of patients were selected in the two groups. The 10 covariates that could affect the surgical outcomes were used to calculate the propensity score, and significant differences in covariates such as age, BMI, extent of surgery, and stage which were observed before the matching were no longer present.

**Table 2:** Baseline characteristics of patients, after propensity score matching

Variables	Open group	Robot group	*P* value
	(*n* = 109)	(*n* = 109)	
Age, years (mean ± SD)	40.81 ± 10.84	41.78 ± 9.36	0.259
Sex, female (%)	91 (83.5)	94 (86.2)	0.700
BMI (median (range))	23.73 (17.58–33.37)	24.53 (16.63–37.77)	0.984
Extent of surgery (total thyroidectomy [%])	93 (85.1)	88 (80.7)	0.278
Lymph nodes dissection			0.940
None (%)	15 (13.8)	14 (12.8)	
Unilateral (%)	72 (66.1)	75 (68.8)	
Bilateral (%)	22 (20.2)	20 (18.3)	
Tumor size (cm, median (range))	0.7 (0.2–2.5)	0.7 (0.2–2.5)	0.331
Multiplicity (%)	25 (22.9)	28 (25.7)	0.760
Bilaterality (%)	20 (18.3)	21 (19.3)	1.000
Extrathyroidal extension (%)	36 (33.0)	38 (34.9)	0.888
Stage (I/III, %)	92 (89.0)/12 (11.0)	93 (85.3)/16 (14.7)	0.317

### Comparison of surgical outcomes

Table 3 shows the comparison of surgical outcomes between the two groups. The operation time was longer in the RT group (*P* < 0.001), and the total amount of hospital cost was higher in the RT group than that in the OT group (*P* < 0.001). There were no significant differences between the two groups in the length of hospital stay (*P* = 0.293) and postoperative pain score (*P* = 0.669). Postoperative complications showed no differences between the two groups (minor complications [*P* = 0.852], major complications [*P* = 0.754]). The number of cases that showed identification of the parathyroid gland with permanent pathology (*P* = 1.000) and the number of parathyroid glands saved during the operation (*P* = 0.160) were not different between the two groups.

**Table 3:** Comparison of the surgical outcomes between two groups, after propensity score matching

Variables	Open group	Robot group	*P* value
	*n* = 109	*n* = 109	
Operation time (min, mean ± SD (range))	107.94 ± 30.84 (60–280)	290.57 ± 74.37 (160–715)	< 0.001
Hospital stay (days, mean ± SD (range))	3.40 ± 1.24 (2–11)	3.56 ± 0.83 (2–8)	0.293
Cost ($, mean ± SD)	2995 ± 695	7,632 ± 1,282	< 0.001
Pain score, operation day (mean ± SD (range))	3.84 ± 1.11 (0–7)	3.76 ± 1.22 (0–7)	0.669
Pain score, postoperative 1 day (mean ± SD (range))	2.36 ± 0.99 (0–5)	2.38 ± 1.06 (0–5)	0.952
Parathyroid gland in pathology	42 (38.5)	41 (37.6)	1.000
Identified number of parathyroid glands during surgery (mean ± SD (range))	2.68 ± 0.88 (0–4)	2.54 ± 0.87 (1–4)	0.160
Minor complication (%)	37 (34)	43 (39)	0.852
Transient hypocalcemia	29	36	
Transient hoarseness	6	7	
Wound seroma	1	0	
Wound infection	1	0	
Major complication (%)	6 (5.5)	4 (3.7)	0.754
Bleeding	1	1	
Chyle leakage	2	0	
Permanent hoarseness	1	1	
Permanent hypocalcemia	2	2	
Radioiodine ablation (%)	52 (47.7)	67 (61.5)	0.033
Stimulated thyroglobulin (ng/mL, median (range))	0.25 (0–66.7)	0.20 (0–6.8)	0.954
No. of metastatic lymph nodes (mean ± SD (range))	1.26 ± 3.07 (0–19)	0.60 ± 1.26 (0–5)	0.133
No. of retrieved lymph nodes (mean ± SD (range))	5.29 ± 5.25 (0–29)	3.50 ± 3.55 (0–17)	0.002

Radioiodine ablation therapy was conducted in 61.5 % of patients in the RT group (67/109 patients) and in 47.7 % of patients in the OT group (52/109 patients). The mean TSH-stimulated Tg level was not different between the two groups (*P* = 0.954).

There was no difference between the two groups in the mean number of metastatic lymph nodes, but the RT group showed less number of retrieved lymph nodes than the OT group (3.50 ± 3.55 [range, 0–17] vs. 5.29 ± 5.25 [range, 0–29]).

## Discussion

We conducted this study to analyze our initial experiences of BABA robotic thyroidectomy for the last 5 years and to compare the surgical outcomes between RT and OT for assessing the feasibility of robotic thyroidectomy for PTC.

In our study, baseline clinicopathologic characteristics were different between the two groups. The RT group showed a lower mean age, lower mean BMI, higher proportion of lobectomy than total thyroidectomy, and lower stage (UICC/AJCC seventh edition), although the tumor size was not different. These differences may be due to a greater desire to avoid a visible anterior neck scar in younger patients, and RT was not recommended in the patients with clinically suspected lymph node metastases. Thus, the findings of this study were inevitably influenced by several confounding factors including a selection bias between the RT and OT groups. The patient’s preferences and narrow indication for RT in our hospital may be the major causes of selection bias. We think that the economic burden of robotic surgery is the main reason why we cannot conduct a randomized study.

The propensity score analysis was used to reduce the confounding factors [27]. Several clinical features and surgical outcomes were compared between the paired 109 patients in both groups after propensity score matching analysis.

The RT group showed a significantly longer operating time. The main contributing factors are the process of creating the flap and robotic docking, which are required for the robotic system operation, and most of the other studies showed similar results [8–24]. But, the robotic operative time is likely to decrease with accumulation of experience and overcoming the learning curve [7].

As expected, the hospital cost in the RT group was about three times higher than that in the OT group. Although we cannot ignore the fact that robotic surgery causes an increase in the total health cost, from a personal point of view, this problem can be solved by lowering the price via competing with other suppliers. In addition, the problem of high cost may be naturally resolved when robotic surgery is popularized like laparoscopic surgery. Most importantly, it can be affordable enough on considering additional excellent cosmetic benefits [10, 11].

On the assessment of safety of RT, there was no significant difference in complication rates between the two groups. This may be an important result on considering the advantage of robotic surgery like fine movement and magnification view. Equivalence of complication rates is enough to demonstrate the safety of RT, considering the low incidence of serious complications after OT.

TSH-stimulated Tg level measured for assessing surgical completeness in papillary thyroid carcinoma was not different between the two groups. TSH-stimulated Tg is one of the important clinical parameters that reflect surgical completeness [30]. The study that particularly analyzed surgical completeness of RT showed a similar result [12], and there is a study that showed superiority of surgical completeness of RT [13]. In this study, while there was no difference in the TNM stage between the two groups, the rates of RAI ablation therapy was higher in the RT group (Table 3). This difference might have resulted from aggressive treatment policy at our institution. We performed RAI ablation therapy in accordance with the ATA guidelines [29] in most cases, but selected patients with stage I disease who received RAI ablation therapy, especially those with angiolymphatic invasion, multifocal disease, nodal disease, and aggressive histology.

There was no difference in the number of metastatic lymph nodes between the two groups, but the number of retrieved lymph nodes was lower in the RT group. To date, there is no consensus about the prognostic implications of lymph node ratio in PTC. The recently published seventh UICC/AJCC staging criteria of thyroid carcinoma do not evaluate lymph node ratio [28]. But, the importance of the LN ratio in PTC has been reported [31–35] and it is likely to have a greater oncological significance in PTC, as in cases of other solid organ cancers [36–38]. Although the follow-up period was short (range, 22–68 months), there was no case of recurrence in the RT group.

Although the absolute value of the retrieved lymph nodes seemed to show a marginal difference, similar results were observed in other studies and meta-analysis [14–16]. The limitation of central node dissection was also reported with a trans-axillary approach [17, 18]. Despite the strong advantages of the robotic arm multi-articulated joint system, directional rigidity of the scope and restricted view of the lower part of the neck are considered to be the most important causes of limitation of central node dissection and it is a matter that needs to be carefully considered as a limitation of RT.

However, several studies reported that the number of retrieved lymph nodes in central node dissection is similar in both robotic and open thyroidectomies [19, 20], and various methods are being attempted to overcome the limitation of the field of view in the lower part of the neck (e.g., make widening the camera view by applying elastic bandage at the lower breast and change the operation table to reverse Trendelenburg position) [15]. The most important part to ensure good visibility of the lower part of the neck is secure sufficient space at the lower neck region during the process of creating the flap.

In our experience, with the sense of incompatibility, the great feature of the robot joint function was not fully utilized at the beginning of robotic surgery. This could simply indicate the learning curve, but familiarity of conventional endoscopic or laparoscopic equipment can act as additional difficulties for expert surgeons. And, the result of this study with a lower number of retrieved lymph node might have been influenced by 30~40 of cases of the early period. It will be explained with further analysis after the experience has accumulated.

Currently, BABA RT has not been accepted as a standard surgical method for PTC, but results of recent studies including a meta-analysis generally show favorable surgical outcomes of RT [21–23]. Accordingly, recent interests are being focused on functional benefits of RT. We analyzed postoperative pain with respect to functional benefit, and there was no significant difference between the two groups. Results for cosmetic satisfaction [10, 11], sensory change in the anterior neck region [24], swallowing discomfort [25], and voice impairments [26] were similar or better with RT compared to OT as well as pain.

## Conclusions

In conclusion, our observational study showed that BABA RT is feasible in terms of surgical safety and surgical completeness that are estimated by postoperative complications and TSH-stimulated Tg, respectively, when compared with OT after adjusting for the selection bias by propensity score matching analysis. But, we could not confirm the oncological safety of BABA RT because it showed limitations in the central compartment LN dissection. In order to establish a clear surgical indication for RT, more evidences are needed that can ensure both surgical and oncological safety.

## Abbreviations

BABA, bilateral axillary breast approach; CND, central node dissection; OT, open thyroidectomy; POD, postoperative days; PTC, papillary thyroid carcinoma; PTH, parathyroid hormone; RAI, radioactive iodine; RT, robotic thyroidectomy; Tg, thyroglobulin
